# Bonding of Composite Cements Containing 10-MDP to Zirconia Ceramics Without Dedicated Ceramic Primer

**DOI:** 10.3290/j.jad.b5362103

**Published:** 2024-05-21

**Authors:** Renato Quirino Ramos, Ben Mercelis, Mohammed H. Ahmed, Marleen Peumans, Guilherme Carpena Lopes, Bart Van Meerbeek

**Affiliations:** a Joint PhD Student at KU Leuven (University of Leuven), Department of Oral Health Sciences, BIOMAT & UZ Leuven (University Hospitals Leuven), Dentistry, Leuven, Belgium, and at UFSC (Universidade Federal de Santa Catarina), Department of Dentistry, Florianópolis, SC, Brazil. Investigation, data curation, visualization, wrote original draft, reviewed and edited the manuscript.; b Lab Coordinator, KU Leuven (University of Leuven), Department of Oral Health Sciences, BIOMAT & UZ Leuven (University Hospitals Leuven), Dentistry, Leuven, Belgium. Investigation, resources, data curation.; c Post-doctoral Research Fellow, KU Leuven (University of Leuven), Department of Oral Health Sciences, BIOMAT & UZ Leuven (University Hospitals Leuven), Dentistry, Leuven, Belgium, and Tanta University, Faculty of Dentistry, Department of Dental Biomaterials, Tanta, Egypt. Statistical evaluation, reviewed the manuscript.; d Professor, KU Leuven (University of Leuven), Department of Oral Health Sciences, BIOMAT & UZ Leuven (University Hospitals Leuven), Dentistry, Leuven, Belgium. Reviewed the manuscript.; e Full Professor, UFSC (Universidade Federal de Santa Catarina), Department of Dentistry, Florianópolis, SC, Brazil. Reviewed and edited the manuscript.; f Full Professor, KU Leuven (University of Leuven), Department of Oral Health Sciences, BIOMAT & UZ Leuven (University Hospitals Leuven), Dentistry, Leuven, Belgium. Conceptualization, reviewed and edited the manuscript.

**Keywords:** zirconia, bond strength, sandblasting, tribochemical silica coating, functional monomer, silane, aging

## Abstract

**Purpose:**

To measure zirconia-to-zirconia microtensile bond strength (µTBS) using composite cements with and without primer.

**Materials and Methods:**

Two Initial Zirconia UHT (GC) sticks (1.8x1.8x5.0 mm) were bonded using four cements with and without their respective manufacturer’s primer/adhesive (G-CEM ONE [GOne] and G-Multi Primer, GC; Panavia V5 [Pv5]), and Panavia SA Cement Universal [PSAu], and Clearfil Ceramic Plus, Kuraray Noritake; RelyX Universal (RXu) and Scotchbond Universal Plus [SBUp], 3M Oral Care). Specimens were trimmed to an hour-glass shaped specimen whose isthmus is circular in cross-section. After 1-week water storage, the specimens were either tested immediately (1-week μTBS) or first subjected to 50,000 thermocycles (50kTC-aged μTBS). The fracture mode was categorized as either adhesive interfacial failure, cohesive failure in composite cement, or mixed failure, followed by SEM fracture analysis of selected specimens. Data were analyzed using linear mixed-effects statistics (α = 0.05; variables: composite cement, primer/adhesive application, aging).

**Results:**

The statistical analysis revealed no significant differences with aging (p = 0.3662). No significant difference in µTBS with/without primer and aging was recorded for GOne and PSAu. A significantly higher µTBS was recorded for Pv5 and RXu when applied with their respective primer/adhesive. Comparing the four composite cements when they were applied in the manner that resulted in their best performance, a significant difference in 50kTC-aged μTBS was found for PSAu compared to Pv5 and RXu. A significant decrease in µTBS upon 50kTC aging was only recorded for RXu in combination with SBUp.

**Conclusion:**

Adequate bonding to zirconia requires the functional monomer 10-MDP either contained in the composite cement, in which case a separate 10-MDP primer is no longer needed, or in the separately applied primer/adhesive.

Zirconia, introduced in dentistry in the early 1990s, initially served as a metal substitute to fabricate frameworks for fixed partial dentures (FPDs) and implants abutments.^16,17,25^^,27,28^ The first generation of zirconia, known as 3Y-TZP, combines zirconium dioxide (ZrO_2_) – doped with 3 mol% yttria (Y_2_O_3_) – with a relatively high alumina content (Al_2_O_3_: ≥0.25 wt%) and other ceramic oxides.^7,10,12^ It is characterized by high strength (800-1000 MPa) and fracture toughness (6 to 8 MPa·m^1/2^), but its opacity limited its use to the posterior region.^7,12^ Additionally, the risk of chipping of the veneering ceramic when using 3Y-TZP as the framework is higher than that of metal framework FPDs.^24,32^ To avoid veneering failures, full-contour zirconia was next manufactured, although its esthetic appeal remained limited as its opacity was still high.^27^ Modifications in the sintering process and composition led to translucent zirconia, broadening its application to the anterior region. However, these translucent monolithic zirconia ceramics exhibited reduced flexural strength and fracture toughness compared to their opaque counterparts.^7,10,18,22,27,35^

Bonding to zirconia has been a topic of investigation since its introduction for dental applications.^17^ Unlike silica-based ceramics, zirconia lacks a glass component, making its surface resistant to etching with hydrofluoric acid and subsequently not receptive for silane coupling for adhesive luting purposes.^11,17^ To overcome this limitation, alternative bonding approaches have been proposed to achieve micromechanical interlocking and chemical bonding between composite cement and zirconia.^11,16,30,33^ Examples of surface pretreatments to enable (micro-)mechanical interlocking include alumina sandblasting (ASB), tribochemical silica coating (TSC), glass-bead air abrasion, diamond and disk grinding, electrical machine discharging, plasma coating, laser irradiation, nanostructured alumina coating, etching with different acidic solutions, and zirconia-ceramic powder coating.^8,18,33^ Surface pretreatments to promote chemical bonding include porcelain coating, selective infiltration etching, silano-pen (pyrosil pen) firing, magnetron-sputtering physical vapor deposition, and zirconia priming with metal primers and silanes.^8,33^

The long-term clinical performance of ceramic restorations hinges on proper adhesive luting procedures.^13^ Two extensively studied zirconia-ceramic surface treatments involve (1) ASB followed by 10-methacryloxydecyl dihydrogen phosphate (10-MDP) primer application, which is contained in different bonding materials such as self-etch primers and adhesives, dedicated zirconia primers, universal adhesives, and composite cements, and (2) TSC followed by a combined 10-MDP/silane primer application, known as “universal restoration” primers.^18,41^ ASB effectively cleanses and microroughens the surface, enhancing surface area, surface energy, and wettability, making the zirconia intaglio surface more receptive to a resin-based bonding agent for micromechanical interlocking. TSC, on the other hand, deposits silica particles onto the surface, enabling subsequent chemical bonding of the composite cement via silane coupling.^8,13,31^ To facilitate and simplify bonding to zirconia, self-adhesive composite cements containing 10-MDP (or other functional monomers) have been developed, with the claim that separate 10-MDP priming is no longer necessary.^34^ Studies have demonstrated that 10-MDP-containing composite cements effectively bond to ASB-treated zirconia surfaces.^11,17,31^ However, the potential additional benefit of using a separate 10-MDP-containing primer prior to the application of a 10-MDP-containing composite cement still needs to be clarified.^33^

The purpose of this laboratory study was to measure the (immediate) microtensile bond strength (µTBS) to two high-translucency zirconia ceramics when using 10-MDP-free or 10-MDP-containing composite cements in combination with or without a 10-MDP-containing primer, followed by an artificial aging protocol to measure the aged µTBS. The null hypotheses tested were that (1) the functional monomer 10-MDP would not be essential for zirconia bonding; (2) a 10-MDP-containing composite cement would not necessitate separate application of a 10-MDP primer to adequately bond to zirconia; (3) aging by long-term thermocycling would not affect zirconia bonding.

## Materials and Methods

One Initial Zirconia Disk UHT (GC; Tokyo, Japan) CAD/CAM block was sectioned into 160 rectangular bars, measuring 2.2 x 2.2 x 6.0 mm, using an automatic precision cutting machine (Accutom 50, Struers; Ballerup, Denmark) under water irrigation. The zirconia bars were sintered following the manufacturer’s instructions (Table 1). After cooling, the zirconia had shrunk by about 16vol%, by which the bars were reduced to a final dimension of 1.8 x 1.8 x 5.0 mm (±0.1 mm). The sintered zirconia bars were ultrasonically cleaned in acetone for 10 min followed by thorough drying with an oil-free air syringe. Then, the zirconia bars were sandblasted for 5 s using a sandblasting device (Rondoflex, Kavo; Biberach, Germany) loaded with 29-µm aluminum oxide (Velopex International; London, UK) at 2 bar (0.2 MPa) pressure, keeping a 1.0-cm distance to the zirconia surface. After sandblasting, the zirconia bars were once more ultrasonically cleaned in acetone for 2 min to remove residual sand particles, followed by thorough drying with an oil-free air syringe.

**Table 1:** Materials used in the study, their composition, application protocol, and batch numbers

Material	Composition	Application protocol (manufacturer’s instructions*)	Batch no.
Initial Zirconia Disk UHT (GC; Tokyo, Japan)	Y_2_O_3_, Al_2_O_3_, SiO_2_, Fe_2_O_3_, HfO_2_	Heat up to 1000°C for 2 h. Heat up to 1450°C for 4.5 h. Hold at 1450°C for 2 h. Cool down from 1000°C for 1 h.	1812101
G-CEM One (GOne) (GC) 10-MDP-containing cement	Paste A: fluoroaluminosilicate glass, UDMA, dimethacrylate, initiator, stabilizer, pigment, SiO_2_, 10-MDP Paste B: SiO_2_, trimethoxysilane, UDMA, 2-hydroxy-1,3-dimethacryloxypropane, 10-MDP, 6-tert-butyl-2,4-xylenol, 2,6-di-tert-butyl-p-cresol, EDTA disodium salt dehydrate, vanadyl acetylacetonate, TPO, ascorbic acid, camphorquinone, MgO	Before the first use, let a small amount bleed out. Attach a mixing tip, extrude a small amount, then apply the material on the zirconia surfaces to be bonded. Lightly press one zirconia specimen to its counterpart. Remove excess cement. Place a glass slide over the specimens, keeping light pressure between the zirconia bars, and light cure for 10 s. Remove the specimen from the mold, and light cure for 10 s from each side.	2010281
Panavia V5 (Pv5) (Kuraray Noritake) Non-10-MDP-containing cement	Paste A: bis-GMA, TEG-DMA, hydrophobic aromatic dimethacrylate, hydrophilic aliphatic dimethacrylate, initiators, accelerators, silanated barium-glass filler, silanated fluoro-alumino-silicate glass filler, colloidal silica. Paste B: bis-GMA, hydrophobic aromatic dimethacrylate, hydrophilic aliphatic dimethacrylate, silanated barium-glass filler, silanated alminium-oxide filler, accelerators, camphorquinone, pigments.	Idem	3N0156
RelyX Universal (RXu) (3M Oral Care; Seefeld, Germany) Non-10-MDP-containing cement	Dimethacrylate monomers, phosphorylated dimethacrylate adhesion monomers, photoinitiator system, amphiphilic redox initiator system, radiopaque fillers, rheological additives and pigments	Idem	7746872
Panavia SA Cement Universal (PSAu) (Kuraray Noritake; Tokyo, Japan) 10-MDP-containing cement	Paste A: 10-MDP, bis-GMA, TEG-DMA, hydrophobic aromatic dimethacrylate, HEMA, silanated barium glass filler, silanated colloidal silica, camphorquinone, peroxide, catalysts, pigments Paste B: hydrophobic aromatic dimethacrylate, silane coupling agent, silanated barium glass filler, aluminum oxide filler, surface treated sodium fluoride, camphorquinone, accelerators, pigments	Idem	BL0037
G-Multi Primer (G-MP) (GC)	Ethanol, γ-MPTS, 10-MDP, MDTP, bis-GMA, TEG-DMA	Idem	2101151
Clearfil Ceramic Primer Plus (CPp) (Kuraray Noritake)	γ-MPTS, 10-MDP, ethanol	Apply a thin layer to the adherent surface of the zirconia bars using a micro-tip applicator. Dry with an oil-free air syringe.	2N0061
Scotchbond Universal Plus (SBUp) (3M Oral Care)	10-MDP, HEMA, dimethacrylate resins, Vitrebond copolymer, filler, ethanol, water, initiators, silanes (γ-MPTES/APTES)	Apply a thin layer to the adherent surface of the zirconia bars using a micro-tip applicator and rub for 20 s. Air-blow gently for 5 s. No light curing.	7769897
29-µm aluminum oxide (Velopex International; London, UK)	Aluminum oxide	Aluminium oxide, titanium dioxide	240322

*When luting zirconia with GOne, the use of G-MP is per manufacturer’s instructions optional; when luting zirconia with Pv5, the use of CPp is recommended by the manufacturer; when luting zirconia with RXu, priming with SBUp is optional per manufacturer’s instructions; when luting zirconia with PSAu, no primer is required per manufacturer’s instructions. UDMA: urethane dimethacrylate; 10-MDP: 10-methacryloyloxydecyl dihydrogenphosphate; bis-GMA: bisphenol A diglycidylmethacrylate; TEG-DMA: triethyleneglycol dimethacrylate, HEMA: 2-hydroxyethyl methacrylate; γ-MPTS: γ-methacryloxypropyltrimethoxysilane; MDTP: methacryloyloxydecyl dihydrogen thiophosphate, γ-MPTES: γ-methacryloxypropyltriethoxysilane; APTES: 3-(aminopropyl)triethoxysilane.

Next, two identically sandblasted zirconia bars were bonded together (sandwich specimens) using one of the four composite cements investigated, with or without additional zirconia pretreatment: (1) G-CEM One (GOne , GC) and G-Multi Primer (G-MP, GC); (2) Panavia V5 (Pv5, Kuraray Noritake; Tokyo, Japan) and Clearfil Ceramic Primer Plus (CPp, Kuraray Noritake); (3) RelyX Universal (RXu, 3M Oral Care; St Paul, MN, USA) and Scotchbond Universal Plus (SBUp, 3M Oral Care); and (4) Panavia SA Cement Universal (PSAu, Kuraray Noritake) and CPp. To assist the bonding procedure, two molds were prepared from a rectangular sample measuring 1.8 x 1.8 x 12.0 mm using a vinylpolysiloxane impression material (Examix NDS regular, GC). The bonding protocol for each material tested is detailed in Table 1.

Before starting the bonding procedure in each group, the light output (1250 mW/cm^2^) of the LED light-curing unit used in this study (SmartLite Pro, Dentsply Sirona; Konstanz, Germany) was verified using a MARC Resin Calibrator (Bluelight Analytics; Halifax, NS, Canada). A total of 20 specimens per group were prepared as follows: (1) two identically pretreated zirconia bars were air dried; (2) the primer or adhesive was applied on the bonding surfaces of the zirconia bars of those groups that received additional pretreatment; (3) the zirconia bars were positioned in the customized mold; (4) the composite cement was applied on the zirconia bar surfaces to be bonded; (5) one zirconia bar was lightly pressed to its counterpart and a glass slide was placed over the specimens, keeping light pressure between the zirconia bars; (6) the composite cement was light cured for 10 s; and (7) the sandwich specimen was finally removed from the mold and light cured for 10 s from each side. After a total light-curing time of 50 s, the bonded specimens were kept dry for 1 h at room temperature prior to trimming at the bonded interface in a standardized way to achieve an hour-glass shaped specimen, whose isthmus was circular in cross-section with a diameter of 1.0 (±0.1) mm, using a customized automated BIOMAT microspecimen former equipped with a regular-grit cylindrical diamond bur (842.314.014, Komet; Lemgo, Germany). All specimens were prepared following the same semi-automated procedure (same bur pressure, bur speed, and time of bur contact to the zirconia surface). A new bur was used for each experimental group (n = 10). The diameter of the “hour-glass” isthmus was measured for each specimen using a stereomicroscope with a grid scale (Leica; Wetzlar, Germany).

After 1-week water storage at 37°C, the specimens were subjected either to immediate μTBS testing to measure the immediate μTBS, or to aging via 50,000 thermocycles prior to being tested to measure the 50kTC-aged μTBS. The microspecimens were fixed to a µTBS testing jig using cyanocrylate glue (Model Repair II Blue, Dentsply-Sankin; Tokyo, Japan) and tested in tension mode at a crosshead speed of 1.0 mm/min using an LRX testing machine (Lloyd; Hampshire, UK) equipped with a load cell of 100 N. The µTBS was calculated in MPa by dividing the imposed force (in N) at the time of fracture by the bonded area (in mm^2^). Specimens that failed before actual testing (pre-test failure [PTF]) were assigned a value of 0.0 MPa and included in calculating the µTBS means.

After µTBS testing, the interfaces of all fractured specimens were examined using stereomicroscopy (Stemi 2000-CS, Zeiss; Oberkochen, Germany) at a magnification up to 50X. The failure modes were categorized as cohesive failure in composite cement, adhesive failure at the composite cement-zirconia interface, or mixed failure (partly involving interfacial failure at least at one of the interfaces).

Representative specimens in each group with a µTBS close to the mean and/or that failed prior to testing (pre-test failures) were selected for ultrastructural characterization using scanning electron microscopy (SEM, JSM-6610LV, JEOL; Tokyo, Japan). Prior to examination, the specimens were sputter coated with gold (40 s, 45 mA; JFC-1300, JEOL).

### Statistical Analysis

Statistical analysis was carried out using Linear Mixed-Effects (LME) statistical modelling with specific contrast (R software v4.0.3, R Foundation for Statistical Computing; Vienna, Austria) to determine statistical differences at a significance level α = 0.05. The random factor applied in the statistical model was the individual zirconia specimen (n = 160). Three variables were identified for the LME statistical model: composite cement with four levels (GOne, Pv5, RXu and PSAu), aging protocol with the two levels (1 week and 50,000 thermocycles), and primer/adhesive application with two levels (no primer and primer). First-, second-, and third-order interactions of all variables were statistically evaluated.

## Results

**Fig 1:**
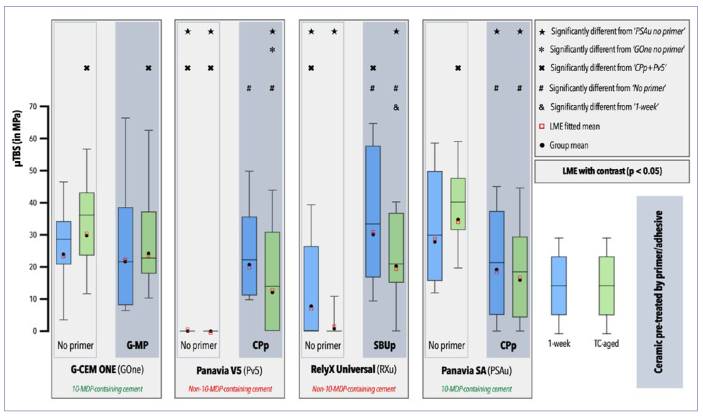
Box-and-whisker plots of the 1-week (blue) and 50kTC-aged (green) microtensile bond strength (μTBS) of the zirconia-bonded sandwich microspecimens.

The µTBS means and fitted LME means are graphically presented in Fig 1 and detailed in Table 2. The statistical analysis of the LME model is presented in Table 3. The first-, second-, and third-order interactions were analyzed, revealing a first-order interaction for the variables composite cement and primer/adhesive application and a second-order interaction for composite cement x primer/adhesive application, meaning that these variables individually or combined influenced the µTBS of the specimens tested. The third-order interaction composite cement x aging protocol x primer/adhesive application was found to be not statistically significant (p = 0.8651), and hence was removed from the statistical LME model.

**Table 2:** Mean microtensile bond strength (µTBS) and fitted LME means for the experimental groups investigated (in MPa)

Experimental groups	Immediate			50kTC-aged		
	Mean µTBS (SD)	PTF	Fitted LME mean	Mean µTBS (SD)	PTF/n	Fitted LME mean
GOne	27.4 (11.7)	0/10	26.6	34.1 (14.9)	0/10	35.0
G-MP+GOne	25.4 (19.3)	0/10	26.3	28.4 (15.6)	0/10	27.5
Pv5	0.0 (0.0)	10/10	0.8	0.0 (0.0)	10/10	-0.8
CPp+Pv5	23.8 (13.3)	0/10	23.1	16.9 (16.1)	3/10	17.6
RXu	9.4 (15.5)	7/10	8.3	1.1 (3.4)	9/10	2.1
SBUp+RXu	34.9 (20.5)	0/10	35.9	23.5 (13.1)	1/10	22.5
PSAu	32.2 (17.6)	0/10	33.3	40.1 (12.2)	0/10	39.0
CPp+PSAu	22.3 (16.8)	2/10	21.2	18.5 (16.2)	2/10	19.6

SD = standard deviation; PTF = pre-test failure; n = number of specimens.

**Table 3:** Linear mixed-effects (LME) statistical analysis for the 1^st^, 2^nd^, and 3^rd^ order interactions (p<0.05)

	Df	F-value	p-value
Composite cement	3	1.778.338	<0.0001*
Aging protocol	1	0.82186	0.3662
Primer/adhesive application	1	666.485	0.0109*
Composite cement x aging protocol	3	225.875	0.0843
Composite cement x primer/adhesive application	3	1.772.407	<0.0001*
Aging protocol x primer/adhesive application	1	262.408	0.1075

* Statistically significant.

Significantly higher µTBS was recorded for Panavia V5 (Pv5) and RelyX Universal (RXu) when applied with their respective primer/adhesive. However, when Panavia SA Cement Universal (PSAu) was applied with its primer, significantly lower µTBS was recorded, while the primer application did not affect the µTBS of G-CEM ONE (GOne). When comparing the composite cements when they were applied in the manner that resulted in their best performance (GOne x G-MP+GOne x CPp+Pv5 x SBUp+RXu x PSAu), the 50kTC-aged groups of the non-10-MDP-containing composite cements (CPp+Pv5, SBUp+RXu) revealed significantly lower µTBS than PSAu, while the 50kTC-aged groups of the 10-MDP-containing composite cements (GOne, G-MP+GOne, PSAu) did not show statistically significant differences among each other. Additionally, the µTBS of 50kTC-aged GOne was significantly higher than that of the 50kTC-aged CPp+Pv5.

Comparing both non-10-MDP-containing composite cements in combination with their respective primer/adhesive (CPp+Pv5 and SBUp+RXu), significantly higher µTBS was found for the SBUp+RXu when tested immediately.

Although the first-order interaction for the variable aging was not significant, meaning that the µTBS was not found to significantly depend on aging, a statistically significant difference was found for SBUp+RXu after 50kTC when compared to the immediately tested specimens (Fig 1).

**Fig 2:**
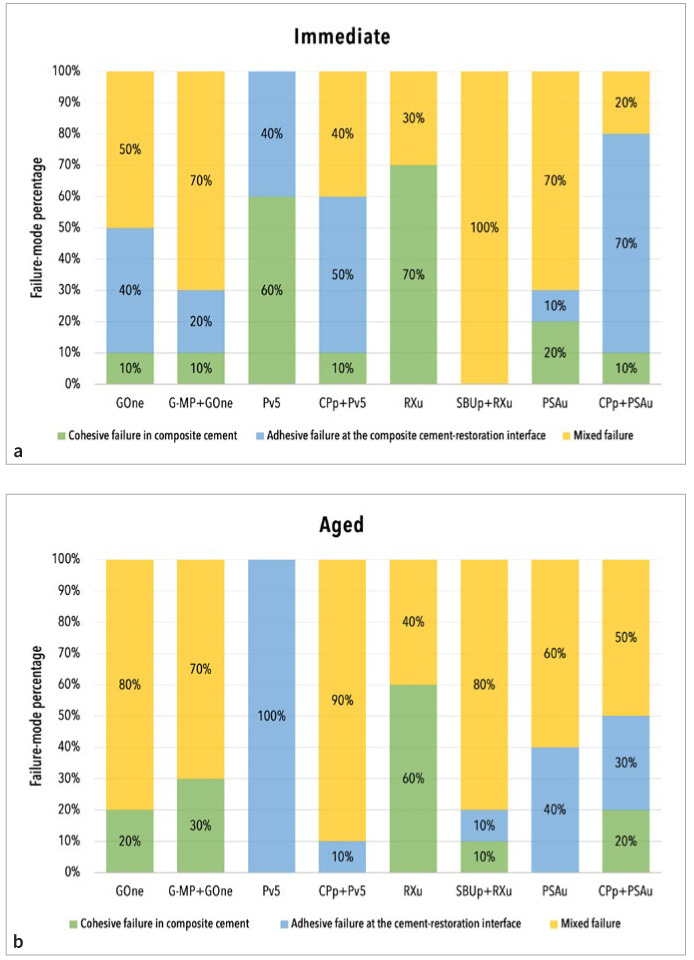
Light-microscopic failure-mode analysis of the zirconia/composite cement/zirconia sandwich specimens tested immediately and after 50,000 thermocycles of aging.

The fracture-mode distribution of all microspecimens is presented in Fig 2. Predominantly mixed failure mode was observed for the majority of the experimental groups (immediately-tested GOne, G-MP+GOne, SBUp+RXu, and PSAu specimens; and 50kTC-aged GOne, G-MP+GOne, CPp+Pv5, SBUp+RXu, PSAu, and CPp+PSAu specimens). Cohesive failure in composite cement of immediately-tested Pv5 and RXu specimens as well as 50kTC-aged RXu specimens, in addition to adhesive failure at the composite cement-zirconia interface of immediately-tested CPp+Pv5, CPp+PSAu specimens and TC-aged Pv5 specimens, were less common. Aging by 50,000 thermocycles had a notable impact on the failure-mode distribution for only three primer/composite cement combinations (Pv5, CPp+Pv5, and CPp+PSAu).

**Fig 3:**
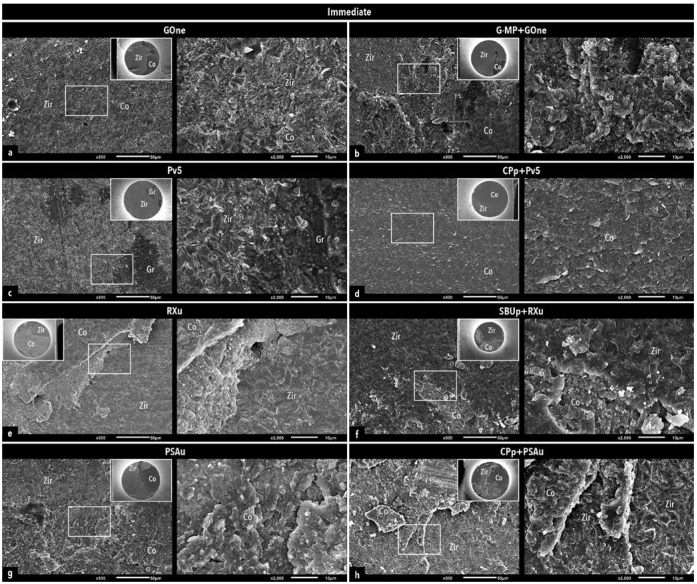
Representative SEM photomicrographs illustrating the predominant failure modes of the experimental groups tested immediately. For each experimental group, an overview image is presented in the small insert in the right top corner, along with a representative area within the white rectangle, which is shown in the adjacent image to the right at a higher magnification. Representative mixed failure for GOne (a) and G-MP+GOne (b); cohesive failure in composite cement for Pv5 (c); adhesive failure at the composite cement-restoration interface for CPp+Pv5 (d); cohesive failure in composite cement for RXu (e); mixed failure for SBUp+RXu (f); mixed failure for PSAu (g); adhesive failure at the composite cement-restoration interface for CPp+PSAu in (h). Co: composite cement; Gr: groove; Zir: zirconia.

**Fig 4:**
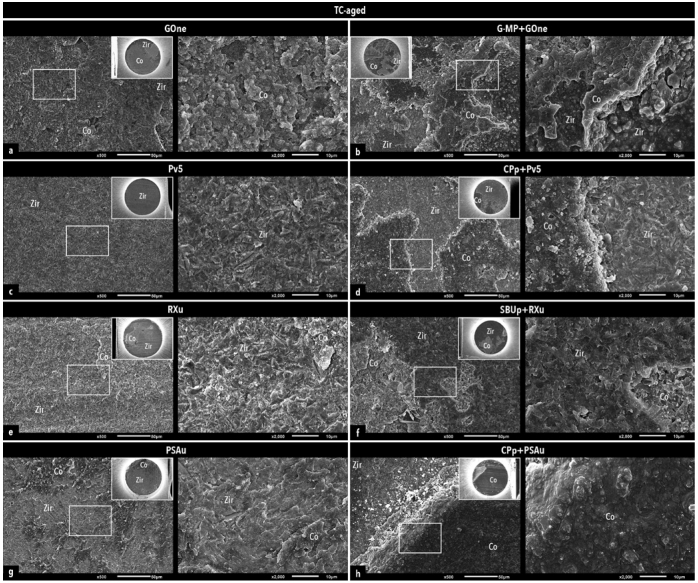
Representative SEM photomicrographs illustrating the predominant failure modes of the experimental groups tested after aging with 50,000 TC. For each experimental group, an overview image is presented in the small insert in the right top corner, along with a representative area within the white rectangle, which is shown in the adjacent image to the right at a higher magnification. Representative mixed failure for GOne (a) and G-MP+GOne (b); adhesive failure at the composite cement-restoration interface for Pv5 (c); mixed failure for CPp+Pv5 (d); cohesive failure in composite cement for RXu (e); mixed failure for SBUp+RXu (f); mixed failure for PSAu (g) and CPp+PSAu in (h). Co: composite cement; Zir: zirconia.

Representative SEM images of fractured microspecimen pairs are presented in Figs 3 and 4, illustrating the predominant failure modes of each experimental group.

## Discussion

This study investigated whether the functional monomer 10-MDP is essential for a durable bond to zirconia. The adhesive luting performance in terms of µTBS of two composite cements that contain 10-MDP (GOne, PSAu) were compared to that of two composite cements that do not contain 10-MDP (Pv5, RXu). All composite cements were tested with and without a dedicated 10-MDP primer (G-MP for GOne; CPp for PSAu and Pv5) or a 10-MDP-containing adhesive (SBUp for RXu), even when not recommended by the respective manufacturer.

The experimental design of this study focused on evaluating bonding of a composite cement to zirconia and did not assess bonding performance to tooth structure. To exclusively measure bond strength to zirconia, zirconia-to-zirconia sandwich microspecimens were prepared instead of zirconia-to-tooth assemblies, thereby eliminating any potential biological tooth-variance effects. However, it is essential to acknowledge that the success of adhesively luting ceramic restorations depends on the bonding performance to both the restoration and the tooth structure.^37^ Furthermore, since different test methodologies might lead to different outcomes, this double bonded interface might have generated different results compared to a single-interface microtensile bond strength test.^26,40^

An hour-glass shape whose isthmus was circular in cross-section was prepared at the interface not only to remove excess composite cement in a consistent and controlled manner, but also to ensure that the tensile stress imposed during testing was concentrated at the (double) interface. For this purpose, a custom-adapted computer-controlled BIOMAT microspecimen former was used to standardize the procedure, ensuring that each specimen received a similar amount of post-bonding specimen-processing stress. Due to zirconia’s high rigidity, a considerable amount of vibration and stress was imposed on the (double) interface of each specimen during processing. This might have increased the occurrence of PTFs as a consequence of post-bonding specimen processing, but otherwise may also be indicative of lower bonding performance. Following the bonding procedures, the specimens were stored dry for 1 h to allow the composite cement to set under optimal conditions before trimming them to achieve an isthmus circular in cross-section.

The µTBS data were analyzed using linear mixed-effects (LME) statistics rather than ANOVA, considering the advantage of LME modelling, the fact that the data were not normally distributed, and that PTFs occurred in certain experimental groups. LME is a robust statistical model, which is more powerful than alternative statistical analyses and also does not require normally distributed data.^36^ The composite cements GOne and PSAu were used as references for the statistical analysis because they revealed the highest µTBS means without the need for application of a 10-MDP-containing primer.

Thermal stress and hydrolysis induced by thermocycling (TC) between 5°C and 55°C can simulate the effect of the varying temperatures in the oral cavity. 10,000 cycles have been claimed to represent about 1 year of clinical function.^14^ In the present study, thermocycling for 50,000 cycles was chosen to challenge the durability of the adhesive zirconia-to-zirconia interface. In fact, specimen aging only had an impact in the group that combined a composite cement lacking 10-MDP with a 10-MDP/silane-containing adhesive (SBUp+RXu). While silane increases surface wettability, it introduces a potential drawback by interfering with the adsorption of 10-MDP molecules onto zirconia, resulting in unreacted silane organic compounds left on the zirconia intaglio surface.^4,20,44^ Furthermore, since silane molecules are susceptible to hydrolytic degradation, their presence on the zirconia intaglio surface may compromise bond longevity.^18,19,23^ Although the µTBS of all tested primer/adhesives that contain both silane and 10-MDP could potentially have been affected by thermocycling for that reason, only SBUp+RXu was affected. While CPp and G-MP contain γ-methacryloxypropyltrimethoxysilane (γ-MPTS), SBUp contains both γ-methacryloxypropyltriethoxysilane (γ-MPTES) and 3-(aminopropyl)triethoxysilane (APTES). Analysis of the interaction between silane and 10-MDP is beyond the scope of this study, but different silanes might react differently with 10-MDP, which could explain why TC aging only affected SBUp+RXu. Furthermore, SBUp’s composition is the most complex of all tested primer/adhesives. Not only silane might have affected µTBS upon aging, but other components might also have done so.

Most strikingly, the composite cement Pv5 – which lacks 10-MDP – failed to bond to zirconia without 10-MDP-containing primer (CPp) application. No µTBS (0.0 MPa) could be measured when tested immediately or upon TC-aging, as all specimens failed during specimen preparation prior to testing (PTFs). Very weak (mean) µTBS (immediate: 9.4 MPa; 50kTC-aged: 1.1 MPa) was recorded for RXu, which lacks 10-MDP, when zirconia was not pre-treated with the adhesive SBUp, which contains 10-MDP. Seven out of 10 immediate microspecimens and 9 out of 10 aged microspecimens failed prior to testing (PTFs). In all experimental groups where 10-MDP was involved, either contained in the composite cement (GOne, PSAu) or when a primer/adhesive containing 10-MDP (G-MP, CPp, SBUp) was applied, an immediate µTBS well above 20 MPa and a 50kTC-aged µTBS above 15 MPa was measured. Hence, the first hypothesis tested that the functional monomer 10-MDP is essential for zirconia bonding, was accepted.

When luting zirconia with the non-10-MDP-containing Pv5, the use of the 10-MDP-containing primer CPp is recommended by the manufacturer; this study confirmed the manufacturer’s recommendation. When luting zirconia with the non-10-MDP-containing RXu, the manufacturers claim that priming with SBUp is optional. However, this study clearly showed that RXu requires the prior application of the universal adhesive SBUp, applied as a primer without light curing, to achieve durable bonding to zirconia. Hence, SBUp priming is mandatory, not optional.

The immediate and 50kTC-aged µTBSs of the 10-MDP-containing composite cements applied without a separate, dedicated 10-MDP primer was not significantly better (GOne) or was significantly better (PSAu) than those recorded when the two composite cements were applied on zirconia pretreated with 10-MDP primer (G-MP and CPp, respectively). The second hypothesis, that a composite cement containing 10-MDP would not require the separate application of a 10-MDP primer to adequately bond to zirconia, was accepted. Indeed, application of the 10-MDP-containing primer G-MP prior to the composite cement GOne had no significantly positive or negative effect on the immediate and 50kTC-aged µTBS. Somewhat unexpectedly, the CPp+PSAu combination revealed a significantly lower immediate and TC-aged µTBS (despite having 2 PTFs out of 10 specimens), when compared to PSAu applied without primer (no PTFs). As the exact content and amount of each component within the primers and composite cements are unknown, a clear explanation for this different material-dependent effect, which may also be based on specific primer/composite cement interactions, cannot be given. However, a factor that might have resulted in the lower µTBS of CPp+PSAu is potential contamination of the zirconia surface with silane, impairing the interaction of 10-MDP with zirconia.^44^ The primers CPp and G-MP are both restoration primers that contain functional monomers to bond not only to zirconia but also to different restorative materials such as glass-ceramics, resin composites, and even metals. While the manufacturer’s technical information on CPp lists 10-MDP and silane (γ-MPTS) in ethanol, G-MP’s composition is more complex, as it not only contains 10-MDP and silane (γ-MPTS) but also 10-methacryloyloxydecyl dihydrogen thiophosphate (MDTP) to enable bonding to (precious) metals and even the cross-linking methacrylate monomer triethylene glycol dimethacrylate (TEG-DMA). Compositional differences may result in different amounts of 10-MDP available to chemically react with the zirconia surface, and thus potentially explain the different bonding performances recorded in this study for the two self-adhesive 10-MDP-containing composite cements GOne and PSAu.^5,18,21,23,44^

Long-term 50kTC-aging only resulted in a significantly lower µTBS for the SBUp+RXu combination, by which the third hypothesis, that aging by long-term thermocycling would not affect zirconia bonding, was only rejected for SBUp+RXu, but not for GOne, G-MP+GOne, CPp+Pv5, PSAu and CPp+PSAu (excluding Pv5 and RXu because of PTFs).

When tested immediately and after 50kTC-aging, overall, the 10-MDP-containing composite cements GOne and PSAu performed best and most consistently without separate zirconia priming, indicating that this simplified zirconia-bonding protocol is a viable alternative to the primer/composite cement combination. The versatile composite cement GOne, according to the manufacturers instructions optionally applicable with or without G-MP, performed most consistently regardless of application mode. Somewhat unexpectedly, no superior adhesive luting performance was recorded for the non-10-MDP-containing composite cements Pv5 and RUn applied following zirconia-surface priming with CPp and SBUp, respectively. Theoretically, a dedicated liquid primer/adhesive is expected to provide better surface wetting, more intensively interact with the surface, and thus promote adhesion better than a more viscous luting composite. 50kTC-aging even significantly reduced the µTBS of SBUp+RXu (1 PTF out of 10 specimens), but not that of CPp+Pv5, despite 3 PTFs out of 10 specimens after 50kTC-aging. Comparing CPp with SBUp, one would expect CPp as a dedicated primer to outperform SBUp, being an adhesive with a complex composition and thus more competition for 10-MDP to interact with the zirconia surface.^17-19,23^ Nevertheless, SBUp+RXu performed as well as CPp+Pv5.

Extensive research has been conducted on zirconia bonding, including systematic reviews and meta-analyses.^2,8,9,11,13,16-19,29-31,33,39,41-43^ A good consensus exists that the zirconia-bonding protocol needs to consist of two main steps: (1) surface cleaning/roughening/pretreatment, followed by (2) the application of a 10-MDP-containing primer/adhesive/composite cement. For surface pretreatment, the two methodologies most described in scientific literature are alumina sandblasting (ASB) or tribochemical silica sandblasting (TCS).^8,16,18,33^ While silane application is not mandatory for bonding to an ABS-treated zirconia surface, for TCS, a silane-containing primer/adhesive is needed.^5,19,44^ In this study, ASB using a relatively small particle size of 29 µm was chosen because it optimally pretreats zirconia for bonding without damaging the intaglio surface of the restoration, as opposed to the use of larger alumina-particle sizes.^8,38^

Irrespective of the zirconia-surface pretreatment chosen, the intaglio surface of the restoration must be clean for resin-based materials to optimally wet the intaglio surface and the 10-MDP molecules to chemically interact with pure zirconia.^3,6,8,9,15^ Ideally, surface pretreatment should be performed after having tried-in the restoration. Thoroughly rinsing the restoration with water to remove saliva, blood, or any other contaminant after the try-in is insufficient.^3,9,15^ Some residue can remain on the restoration’s intaglio surface, preventing the 10-MDP functional monomers from directly interacting with pure zirconia.^9^ Upon restoration try-in, the best method to clean the intaglio surface of contaminated zirconia restorations is air abrasion with alumina.^2,6,9,15,39^ When contaminated zirconia restorations were alumina sandblasted, the original (non-contaminated) bond strength was restored.^1,9,39,13,15^ Alternative surface-decontamination protocols for ASB involve the use of cleaning agents, such as Ivoclean (Ivoclar; Schaan, Liechtenstein), Katana Cleaner (Kuraray Noritake), and ZirClean (Bisco; Schaumburg, IL, USA). These agents chemically decontaminate the surface through different mechanisms. Ivoclean (Ivoclar) is an alkaline cleaner that contains a potent base (NaOH) in a hypersaturated solution of zirconia particles. It works by creating a greater concentration gradient of zirconia, inducing the phosphates that are bonded to the intaglio surface of the restoration to bond to the zirconia in the cleaning solution. Furthermore, the alkaline agent promotes decontamination by neutralizing and removing other organic contaminants. Katana Cleaner (Kuraray Noritake) contains triethanolamine and a 10-MDP salt as active components. Triethanolamine is a surfactant, whereas the 10-MDP salt is supposed to attach to and encapsulate organic residues, which will subsequently be washed away when the surface is rinsed with water. ZirClean (Bisco) is a highly alkaline cleaner composed of potassium hydroxide (KOH), which breaks the ionic bonds formed between the contaminants and the zirconia surface.^3,41^ Ivoclean, Katana Cleaner, and ZirClean were shown to improve the bond strength to previously contaminated zirconia surfaces; however, their bond strengths were lower than those to non-contaminated zirconia surfaces.^1,3,6,13,15,39,41^ The use of sodium hypochlorite (NaOCl) followed by thorough water rinsing is another decontamination method suggested in the literature as being as effective as ASB.^9,13,39,41^ Extensive water rinsing is recommended after the application of NaOCl to ensure complete removal of the solution, which otherwise may impair polymerization of the composite cement due to oxygen generation.^13,41^

After surface pretreatment and cleaning, the next step in zirconia bonding involves the application of a 10-MDP-containing primer/adhesive/composite cement. As shown in this study, this step is essential to achieve strong, durable bonding of a resin-based luting composite to a zirconia surface.^17,31^ This study demonstrated that even just using a 10-MDP-containing composite cement without any additional 10-MDP-containing primer/adhesive sufficed to effectively bond to zirconia. The importance of 10-MDP in this bonding process has been highlighted in various studies, systematic reviews, and meta-analyses.^8,11,17,29,31,33^ However, this study did not compare different zirconia surface pretreatments. It would be important to evaluate whether different surface pretreatments could lead to findings different from those of this study. If so, the combination of different surface pretreatments with a 10-MDP-containing composite cement could potentially promote even higher bonding effectiveness.

## Conclusion

Durable bonding to zirconia requires the functional monomer 10-MDP, either contained in the restoration primer/adhesive or in the composite cement.

## Clinical relevance:

The functional monomer 10-MDP, either contained in the restoration primer/adhesive or within the composite cement, is needed to durably bond to dental zirconia ceramics.
